# Polarization invariant plasmonic nanostructures for sensing applications

**DOI:** 10.1038/s41598-017-08020-y

**Published:** 2017-08-08

**Authors:** Landobasa Y. M. Tobing, Geat-Yee Goh, Aaron D. Mueller, Lin Ke, Yu Luo, Dao-Hua Zhang

**Affiliations:** 1 Nanophotonics Lab, School of EEE, OPTIMUS, Nanyang Technological University, 50 Nanyang Avenue, Singapore, 639798 Singapore; 2 Institute of Material Research and Engineering, Agency of Science, Technology (A*Star), 3 Research Link, Singapore, 117602 Singapore

## Abstract

Optics-based sensing platform working under unpolarized light illumination is of practical importance in the sensing applications. For this reason, sensing platforms based on localized surface plasmons are preferred to their integrated optics counterparts for their simple mode excitation and inexpensive implementation. However, their optical response under unpolarized light excitation is typically weak due to their strong polarization dependence. Herein, the role of rotational symmetry for realizing robust sensing platform exhibiting strong optical contrast and high sensitivity is explored. Specifically, gammadion and star-shaped gold nanostructures with different internal and external rotational symmetries are fabricated and studied in detail, from which their mode characteristics are demonstrated as superposition of their constituent longitudinal plasmons that are in conductive coupling with each other. We demonstrate that introducing and increasing internal rotational symmetry would lead to the enhancement in optical contrast up to ~3x under unpolarized light illumination. Finally, we compare the sensing performances of rotationally symmetric gold nanostructures with a more rigorous figure-of-merit based on sensitivity, Q-factor, and spectral contrast.

## Introduction

An optics-based sensing platform generally consists of an optical cavity system that is susceptible to external changes in its environment, with its performance typically gauged by the shift of resonance wavelength over the change in refractive index. An ideal sensing platform should be simple in its implementation, robust in its resonance mode excitation, efficient in its strong optical responses, and measurable using inexpensive equipment. One scheme is based on refractive index sensing, which has been extensively investigated in various optical systems^1–11^. Specifically, gold-based localized surface plasmon resonance (LSPR) has attracted much attention in recent years^12–23^ for its strong light localization on metal-dielectric interface and the compatibility of gold with specific protein binding for label-free biosensing^24^. One of the practical advantages of using LSPR for sensing lies in its mode excitation under normal light illumination in microscope setting, which is much simpler than its integrated optics counterparts which require phase matching and high precision optical alignments^25–27^. As the resolution of refractive index sensing depends on the resonance Q-factor, many efforts have been dedicated to produce narrow linewidth resonance in variety of coupled-resonator configurations^17, 28–34^. This so-called Fano resonance results from specific interactions between cavity modes, which are achieved through specific incidence angle or incident polarization. As Fano resonance depends strongly on the spectral overlap and damping factor of the two resonance modes, it typically suffers from low spectral contrast despite its much higher Q-factors compared to isolated metal resonators. From a practical perspective, uncoupled metal nanostructures are still preferred as sensing platform because of their simple mode excitation.

**Figure 1:**
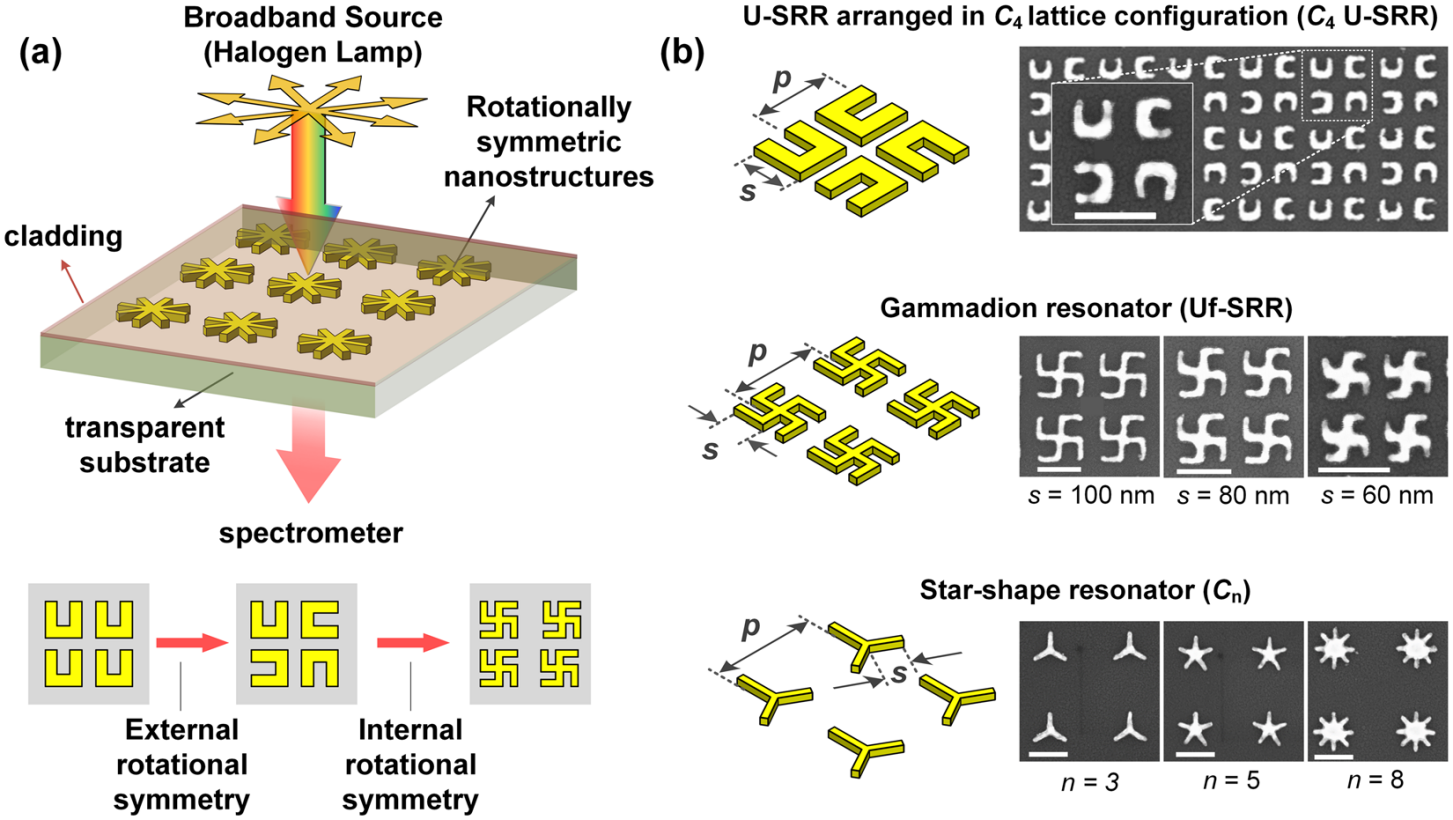
Incorporating rotational symmetry for making robust plasmonic sensing platform. (**a**) Schematic of polarization-invariant sensing platform based on unpolarised broadband light source and rotationally symmetric metal nanostructures. (**b**) Plasmonic nanostructures with varying rotational symmetry. (Top) U-SRRs in *C*_4_ lattice configuration with the periodicity normalized to resonator size, i.e., *p* = 2 *s*. The unit cell is denoted by the dashed line. (Middle) Gammadion structures with periodicity (*p*) normalized to their arm lengths (*s*), i.e., *p* = 3 *s*. (Bottom) Star-shape structures with different rotational symmetry at fixed periodicity of *p* = 400 nm and arm length of *s* = 100 nm. Both the gammadion and star-shaped nanostructure are in square lattice configuration. All scale bars represent 200 nm.

Despite their high sensitivities in sensing, plasmonic resonators typically have weak optical response under unpolarized light illumination due to their strong polarization-dependent responses. This polarization dependence can be overcome by incorporating rotational symmetry into the optical cavity system, either through the lattice configuration (i.e., external rotational symmetry) or the resonator motifs (i.e., internal rotational symmetry). The role of external rotational symmetry has been investigated previously, where we demonstrated stronger optical response in split ring resonators (SRR) arranged in a fourfold rotationally symmetric configuration as compared to that in *u*-shaped SRRs arranged in a typical square lattice^35^. Naturally, a stronger optical response would be expected with higher rotational symmetry, but this cannot be achieved by external rotational symmetry alone as it will be limited to the complexity of the lattice configuration. Here, we further investigate the roles of rotational symmetry in rotationally symmetric structures and evaluate their performance as sensing platform (see Fig. 1a). Using longitudinal plasmon as our analytical framework, the resonance modes of the rotationally symmetric structures can be expressed as a superposition of longitudinal modes from the constituent gold nanorods. The rotationally symmetric structures are also shown to exhibit hybrid magnetic-electric optical responses which depend on specific interactions among individual gold nanorods. Finally, we present the experimental sensing performances of these rotationally symmetric structures in terms of their sensitivities, Q-factors, and spectral contrasts.

## Results

In order to decouple the effects of internal and external rotational symmetries, we fabricated the rotationally symmetric nanostructures together with split ring resonators on the same chip (Fig. 1b), namely the gammadion (Uf-SRR), star-shaped nanostructures (*C*_n_), and the *u*-shaped SRR arranged in fourfold rotationally symmetric lattices (*C*_4_ U-SRR). The *C*_4_ U-SRR structures (top panels) exhibit fourfold external rotational symmetry from their lattice configuration, while the gammadion (middle panels) exhibit both external and internal fourfold rotational symmetry. Both *C*_4_ U-SRRs and gammadions work based on *C*_4_ symmetry. In star-shaped nanostructures (bottom panels), on the other hand, we can have higher internal rotational symmetry associated with the number of arms (*n* > 4). But this is realized at the absence of external rotational symmetry except in few cases (i.e., *n* = 4, 8,.,4*k*). In what follows, we analyse the resonance characteristics of gammadion and star-shaped nanostructures before evaluating their performance as sensing platforms.

### Gammadion structure (Uf-SRR)

**Figure 2:**
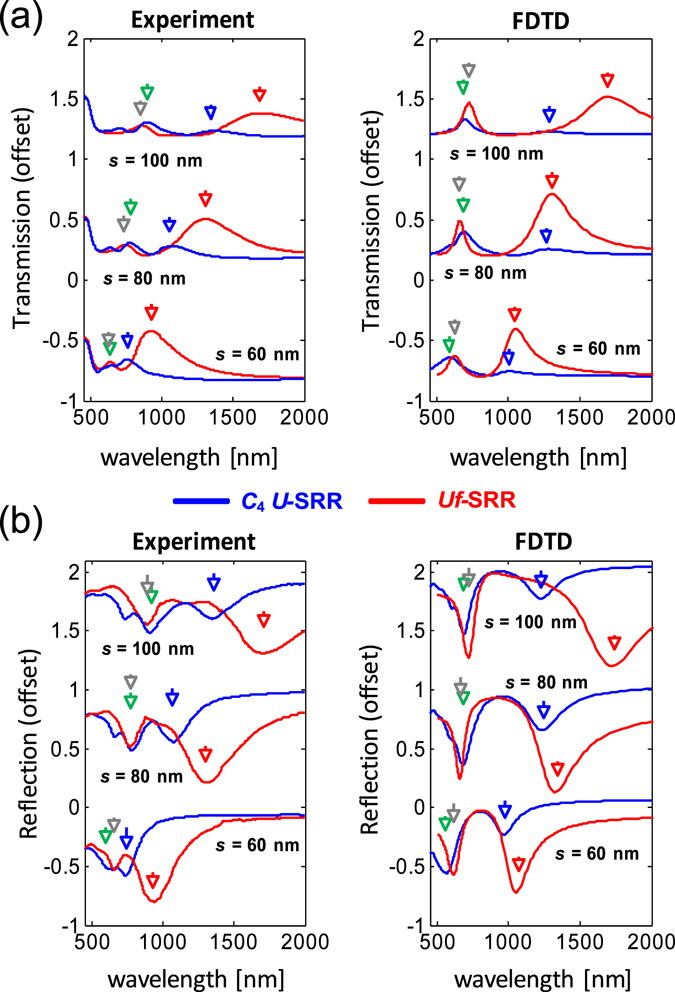
Effect of internal rotational symmetry in improving the optical response under unpolarized light illumination. Transmission (**a**) and reflection (**b**) spectra of the Uf-SRRs (red) and *C*_4_ U-SRRs (blue) for s = 60 nm, 80 nm, and 100 nm, where the measurements and simulations are presented side by side. The arrows denote the modes of the *C*_4_ U-SRRs (green, blue) and the gammadions (red, gray).

The effect of introducing internal rotational symmetry is illustrated in Fig. 2, which compares the measured optical responses of gammadion and *C*_4_ U-SRRs under unpolarized light normal incidence. The resonator size (*s*) of the *C*_4_ U-SRRs was varied from *s* = 60 nm, *s* = 80 nm, and *s* = 100 nm, with the periodicity (*p*) normalized to their sizes, i.e., *p* = 2 *s*. Likewise, arm length of the gammadions (*s*) was varied from *s* = 60 nm, *s* = 80 nm, and *s* = 100 nm, with the periodicity (*p*) normalized to their armlengths, i.e. *p* = 3 *s*. The resonances are identified by the spectral dips (for transmission) and peaks (for reflection), and the resonance positions are indicated by the markers. The gammadions clearly exhibit much stronger optical responses than those of the *C*_4_ U-SRRs, indicating that incorporation of internal rotational symmetry leads to higher spectral contrast. In our previous work^35^, we introduced external *C*_4_ symmetry and observed significant spectral contrast enhancement. Here, the Uf-SRR exhibits ~3x higher spectral contrast with ~2x higher reflection than those of *C*_4_ U-SRRs. However, the resonance modes appear to significantly red shift from the original resonance mode. From their geometry, Uf-SRRs can be understood either as *C*_4_ U-SRRs that are fused together or as two orthogonal *s*-shaped nanorods fused at their center. These two perspectives can be used to better understand the working mechanism of a gammadion.

**Figure 3:**
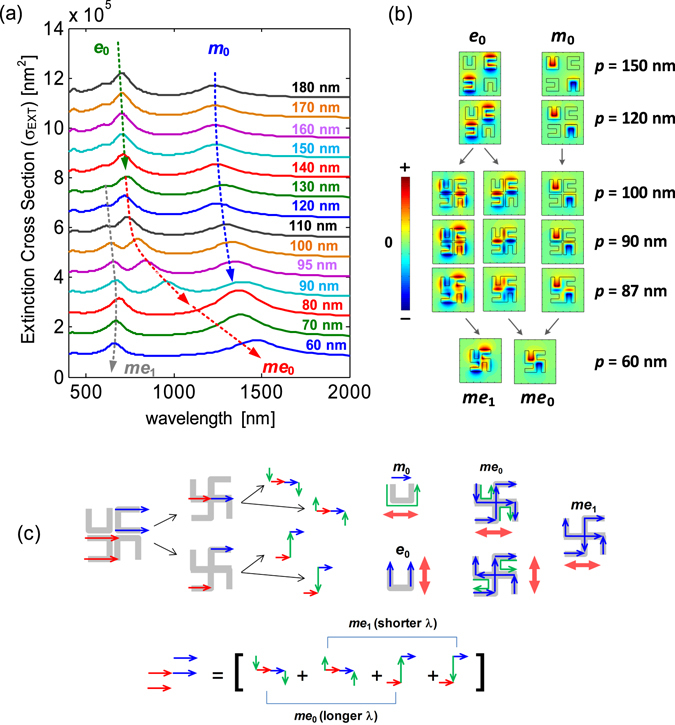
Evolution of modes as the *C*_4_ *u*-SRR changes shape into the gammadion (Uf-SRR). (**a**) The extinction cross section for *C*_4_ U-SRR as the periodicity is varied from *p* = 180 nm to *p* = 60 nm, where the modes changes from those of the SRR (*m*_0_, *e*_0_) into those of the gammadion (*me*_0_, *me*_1_) (as denoted by the dashed lines). (**b**) The *H*_z_-fields at peak positions near *m*_0_ and *e*_0_ modes for different periods. (**c**) Fundamental and higher order longitudinal plasmon excitations in the conductively coupled U-SRRs. The fundamental order plasmons turn into the *me*_0_ mode, while the higher order plasmons turn into *me*_1_ mode.

Gammadion structures have been investigated for their chiral properties^18, 36–38^ and their potential application in detecting left-handed and right-handed molecules^18, 37^. In this work, we instead see the gammadion from the perspective of *C*_4_ U-SRRs, where the spacing between the neighbouring SRRs is reduced until the SRRs are fused together to form a gammadion. As illustrated in Fig. 3a, which shows the extinction cross sections of *C*_4_ U-SRRs at decreasing spacing, we have a situation where the coupling mechanism changes from near-field coupling into conductive coupling. Using the same notations from our previous works^39, 40^, we indicate the magnetic (*m*_0_) and electric (*e*_0_) modes of an SRR with their associated *H*_z_-field distributions in Fig. 3b. At decreasing spacing, one can see that the *m*_0_ mode vanishes while the *e*_0_ mode splits into two resonances that eventually become the fundamental and higher order modes of the gammadion (*me*_0_ and *me*_1_). Note also that the *H*_z_-field distribution of *me*_0_ resembles that of the *m*_0_ but at longer resonance wavelength. The same is observed for the field distributions of *me*_1_, which resembles that of the higher order magnetic resonance (*m*_1_)^35^.

The resonance splitting can be understood as follows. Normally, metallic resonators interact through near-field coupling, such as transverse and longitudinal dipolar couplings. The coupling of electric dipoles is understood as capacitive coupling, while the coupling of magnetic dipoles is understood as inductive coupling. In terms of interaction energy, the transverse coupling results in a blue shift, while the longitudinal coupling results in a red shift^41^. There also exists a conductive coupling in which the conduction current is redistributed when the metallic resonators are contiguous, which eventually enables the indirect excitation of electric dipoles that previously could not be excited under normal incidence. This situation is illustrated in Fig. 3c (upper panel), where electric dipoles associated with *e*_0_ modes interact conductively, resulting in the excitation of electric dipoles in the other part of resonator. As electric dipoles must be conserved in this part of resonator, electric dipoles in opposite directions are thus excited, making the coupled *e*_0_ modes expressible as a superposition of 4 electric dipoles. These electric dipoles are in fact the longitudinal plasmon modes of *s*-shaped nanorods (Fig. 3c, lower panel). The fundamental longitudinal modes correspond to the first and third terms, while the higher order longitudinal modes correspond to the second and fourth term. As these longitudinal modes have different resonance wavelengths, the fundamental modes (first and third term) and the higher order modes (second and fourth term) combine to give *me*_0_ and *me*_1_ modes, respectively.

**Figure 4:**
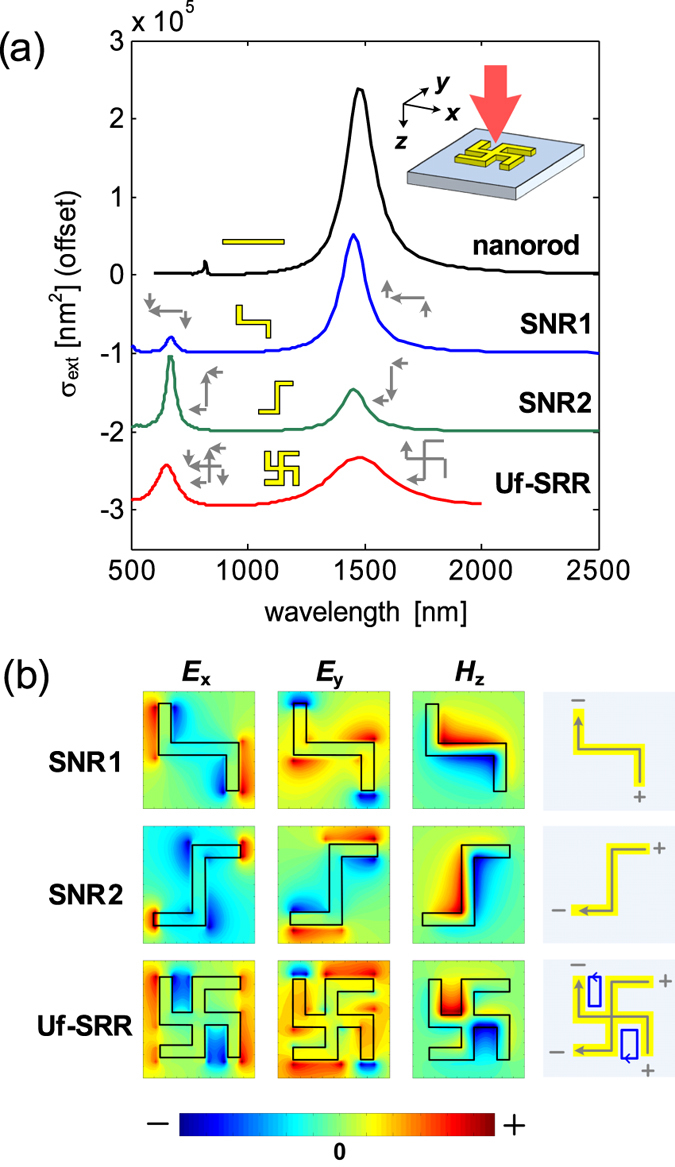
Gammadion (Uf-SRR) as a superposition of two orthogonal *s*-shaped nanorods (SNR). (**a**) The extinction cross sections of a nanorod (NR), SNR1, SNR2, and Uf-SRR for the x-polarized light illumination. The arm length of the Uf-SRR is *s* = 60 nm, and the length of the nanorod is adjusted to have the same total length of the SNR, i.e., *L* = 4 *s*. (**b**) The electromagnetic field distributions of the *S*NRs and Uf-SRR with the indicated electric dipoles (gray) and circulating currents (blue).

To confirm if these are indeed the longitudinal plasmons, we show the calculated extinction cross sections of Uf-SRR and *s*-shaped nanorods (SNR) in Fig. 4a, where the case of a single nanorod (NR) is also presented as a reference. To match the resonance of the NR with that of the SNR, the length of the NR was made the same as the total length of the SNR, i.e., *L* = 4 *s*. This appears to be the case as the extinction cross sections of NR, SNRs, and Uf-SRR exhibit the same resonance wavelengths for the same *x*-polarized light illumination. In addition, there always exist two resonance modes for the SNR with their resonance strengths dependent on the orientation of the SNR with respect to the incident polarizations. This further confirms the existence of the same fundamental and higher order longitudinal plasmons discussed earlier. The electromagnetic field distributions around *me*_0_ resonance for the two SNRs and the Uf-SRR are presented in Fig. 4b. From the *H*_z_-field distribution, we can see that it is possible to excite electric dipole orthogonal to the incident polarization, which is mainly attributed to the conductive coupling of orthogonal s-shaped nanorods. As these two orthogonal SNR are conductively coupled to make Uf-SRR, we can see that the electromagnetic field distribution of the Uf-SRR is also the superposition of the electromagnetic fields associated with each SNR. The combination of electric dipoles from the two SNRs also leads to the generation of the circulating current that produces localized *H*_z_-field similar to the magnetic modes in U-SRRs. It is interesting to note here that a magnetic response can be achieved by a set of nanorods whose resonance characteristic is electric. For this reason, we refer the resonance mode of the Uf-SRR as hybrid magnetic-electric mode as it has strong magnetic response with electric resonance characteristics. This is also the main reason for the observed red shift for Uf-SRR in Fig. 2 because the resonance characteristics of Uf-SRR are determined mainly by the property of the constituent SNRs. As the total length of SNR (*L* = 4 *s*) is longer than that of the U-SRR (*L* = 3 *s*), then the resulting resonance wavelength is longer than that of the *C*_4_ U-SRR.

### Star-shaped nanostructure (C_n_)

**Figure 5:**
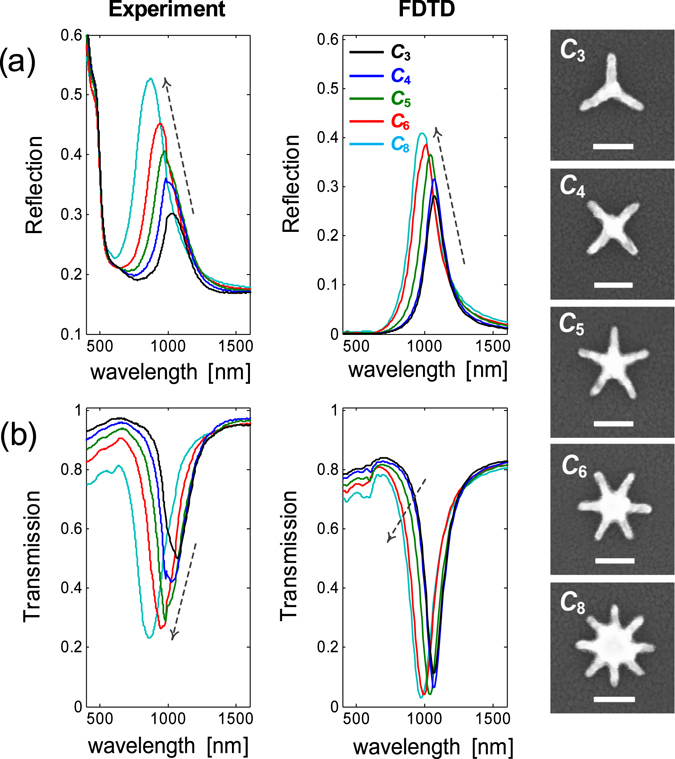
The role of internal rotational symmetry in improving optical response. The transmission and reflection contrasts increase progressively as the rotational symmetry is varied from *n* = 3 to *n* = 8. The arm length (s) and periodicity (*p*) of the *C*_n_ structure is fixed to s = 100 nm and *p* = 300 nm, respectively. Due to fabrication non-idealities in denser structures, the effective arm length is shorter for *C*_n_ with higher rotational symmetry. The scale bars in all the insets represent 100 nm.

We have shown that introducing internal rotational symmetry into an externally rotationally symmetric system leads to ~3x spectral contrast enhancement at the expense of longer resonance wavelength. Here, we investigate the effect of increasing internal rotational symmetry in the star-shaped nanostructures. The star-shaped gold nanoparticles are normally realized through self-assembly methods^15, 42^, with the purpose of making surface-enhanced Raman spectroscopy (SERS) substrate. However, the lack of dimensional control in self-assembly process has hindered the detailed study of the resonance modes of star-shaped nanostructures. We systematically fabricated the star-shaped gold nanostructures with varying internal rotational symmetry from three-fold (*n* = 3) to eight-fold (*n* = 8) at different periodicities. The working mechanism of *C*_n_ structures is also analyzed from the same longitudinal plasmon perspective, where in this case the *C*_n_ consists of *n* gold nanorods connected at the centre of the *C*_n_ structure. The measured optical responses of *C*_n_ structure for increasing *n* is shown in Fig. 5, where the optical response becomes stronger at increasing rotational symmetry. The reflection peak increases from ~0.3 (*n* = 3) to ~ 0.53 (*n* = 8), and blue shifts from ~1071.43 nm (*n* = 3) to ~864.06 nm (*n* = 8). In principle, there is transverse dipolar coupling between the nanorods but such coupling does not contribute significantly to a blue shift in the resonance wavelength (the numerical calculations of *C*_n_ structure seem to confirm this finding, as shown in Fig. S1). However, the increase of transverse coupling (by increasing the number of arms) does contribute to the resonance broadening, leading to lower Q-factor. The larger blue shift in our experiments was attributed to the fabrication non-idealities, which result in higher effective dose around the pattern and in turn affect the geometrical features of the resonator. This is evidenced by the increased “intersection area” and decreased arm length of *C*_n_ structures having larger *n* (see the insets of Fig. 5).

**Figure 6:**
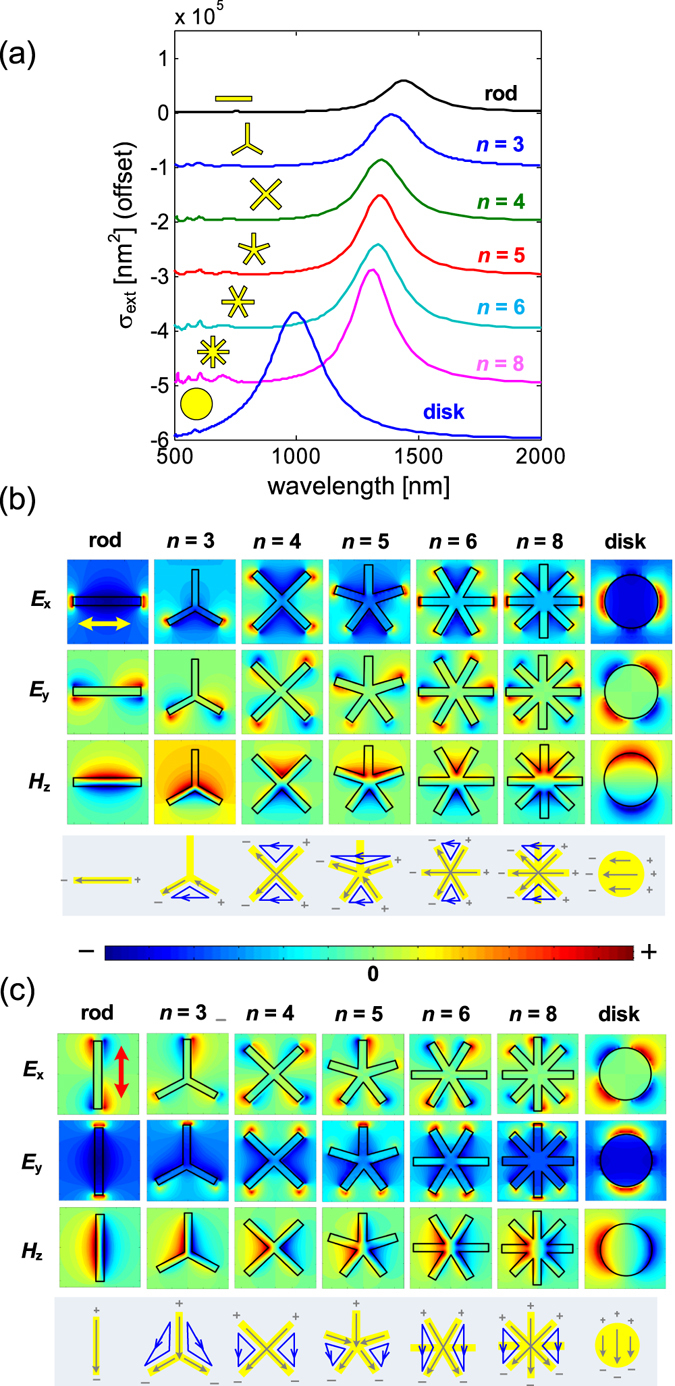
Evolution of modes from a nanorod, to *C*_n_ structures (*n* = 3–8), and finally to nanodisk under *x*-polarized incidence. (**a**) The extinction cross sections of a nanorod, *C*_n_ structures, and nanodisk under unpolarized light illumination. The corresponding electromagnetic field distribution under (**b**) *X*-polarization and (**c**) *Y*-polarization, with the indicated circulating currents (blue), electric dipoles (gray), and incidence polarizations (double arrows). The arm length for all *C*_n_ structure is *s* = 125 nm.

The resonance mode mechanism of *C*_n_ structure is further studied by comparing the calculated extinction cross sections of *C*_n_ structures with those of nanorod (NR) and nanodisk (ND) (see Fig. 6). The same as the Uf-SRR case, where the longitudinal plasmon oscillates back and forth along the *C*_n_ arms, the length of the nanorod was chosen as *L* = 2 *s*. Indeed, the resonance characteristics of *C*_n_ structures are closer to those of the nanorod than the nanodisk (see Fig. 6a). This is as expected from a longitudinal plasmon perspective, where the resonance property of the *C*_n_ structure mostly follows the optical property of the constituent individual nanorods. The mapping of the resonance wavelength of the *C*_n_ structures under different incident polarization angles (see Fig. S1, Supplementary Information) reveals that the polarization invariance occurs only for even number of arms. This is attributed by the role of the rotational symmetry in distributing the conduction current under *x* and *y* polarizations. When *n* is even, the conduction current in the constituent gold nanorod is always equal for both polarizations as the number of arms are equal at both sides. However, the conduction current is not uniformly distributed when *n* is odd. When the incident polarization is along one of the constituent nanorods, for example, there is an extra one arm on one side of the structure which makes the conduction current larger on the side with fewer numbers of arms. At incident polarization perpendicular to one of the constituent nanorods, only *n* − 1 arms are plasmonically active, and the conduction current is equally distributed just like in the even *n* case. The current distribution is expected to be more symmetric as *n* is increased, translating to smaller variations of resonance positions.

The electromagnetic field distributions as the geometry is varied from a nanorod to nanodisk with *C*_n_ structures in between are shown in Fig. 6b (for *x*-polarization) and Fig. 6c (for *y*-polarization). In the NR case, for the *x*-polarization, we have a typical electric resonance associated with electric dipole oscillation along the *x*-direction. As the structure becomes *C*_3_, the arm perpendicular to the incidence polarization is not excited, making the *C*_3_ act as a *v*-SRR with localized magnetic fields in the gap opening formed by the other two arms. When the incidence light is y-polarized, the arm parallel to the y-axis is excited and induces dipole excitation in the other two arms due to conductive coupling. This leads to the generation of circulating currents and localized magnetic fields as illustrated in Fig. 6c (for the *C*_3_ structure). In the *C*_4_ structure, for *x* and *y* polarizations, we have localized magnetic fields in the two gap openings that are opposite each other. In this situation, the resonance characteristic of the *C*_4_ structure is mainly magnetic. However, at 45-degree (or 135-degree) polarization, only one nanorod is excited as the other nanorod is exactly perpendicular to the incident polarization. The main resonance characteristic of the *C*_4_ structure at 45-degree polarization is thus electric in this case. Unlike in the *C*_3_ structure which has hybrid magnetic-electric properties, there is no indirect dipole excitation resulting from the conductive coupling in the *C*_4_ structure. This makes the *C*_4_ geometry qualitatively different from the other *C*_n_ structures, as it exhibits magnetic response under *x* and *y* polarizations, and electric response under 45 degree polarizations. This is illustrated in the mapping of the resonance wavelength under different incident polarization angles (see Fig. S1), whereby the λ_R_ for *n* = 4 clearly deviates from the systematic blue shift for increasing *n*.

In the *C*_5_ structure, as with the *C*_3_ structure, there are two different *v*-SRRs excited under x-polarization, and the arm perpendicular to the x-direction is not excited. The conducting current is redistributed in the same way as in *C*_3_ structure, leading to localized magnetic fields in the other two gap openings (Fig. 6c, *C*_5_ structure). Following the same reasoning for the *C*_6_ structure, two identical *v*-SRRs and one NR are excited under *x*-polarization, and two identical *v*-SRRs (with larger opening angles) are excited under *y*-polarization. In the *C*_8_ structure, we have the same optical responses under *x*-polarization and *y*-polarization, where two *v*-SRRs and one NR are excited. Finally, in the nanodisk structure, we have optical responses similar to that of the nanorod with the resonance wavelength significantly blue shifted from the NR and *C*_n_ structures. For the *C*_n_ structure, note that the localized magnetic fields in some gap openings under *x*-polarization are complemented by those under y-polarization, rendering the *C*_n_ structure suitable for unpolarized light illumination as all the gap opening areas are always activated under any incidence polarization angle. More light localization in the gap openings is the reason for the stronger optical response in structures with higher rotational symmetry, which is also what were observed experimentally in Fig. 5.

## Discussion

Prior to evaluating the sensing parameters of the gold nanostructures considered in this work, it may be useful to gain some insights from the *LC* model^43^. Using the U-SRR for simplicity, we can express the SRR gap capacitance (*C*_SRR_) and SRR inductance (*L*_SRR_) generically as *C*_*SRR*_ = ε_0_ε_d_ *wt*/*g* and *L*_SRR_ = μ_0_ *s* ^2^/*t*, where *ε*_0_ (*μ*_0_) is the electric permittivity (magnetic permeability) in vacuum, *ε*_d_ is the effective relative permittivity of the dielectric medium, *s* is the SRR size, *t* is the SRR thickness, *w* is the width of the SRR arms, and *g* is the SRR gap opening. It can thus be inferred that the change in refractive index would only translate to the change in the SRR capacitance^44^. The resonance wavelength λ_R_ is ${\lambda }_{R}=2\pi c\sqrt{{L}_{SRR}{C}_{SRR}}\propto s\sqrt{{\varepsilon }_{d}}$, showing that the resonance wavelength is linearly proportional to resonator size and the medium refractive index. The sensitivity is defined as ${\rm{\Gamma }}=d{\lambda }_{R}/d(\sqrt{{\varepsilon }_{d}})={\lambda }_{R}/\sqrt{{\varepsilon }_{d}}\propto {\lambda }_{R}$, indicating that the sensitivity depends mainly on resonance wavelength regardless of the geometrical shapes. Note that higher sensitivity is also expected when the resonators are placed on lower effective permittivity ε_d_, which can be achieved in lower refractive index substrate or by fabricating the resonators on a pedestal (free-standing)^3, 19^. The figure-of-merit (FoM), on the other hand, can be expressed as^45^ ${\rm{FoM}}={\rm{\Gamma }}/{\rm{\Delta }}\lambda ={\rm{\Gamma }}Q/{\lambda }_{R}\propto Q$, suggesting that FoM is only dependent on the resonance *Q* factor. It is possible to derive the Q-factor by incorporating the resistance element into the LC circuit, where the resistance may consist of radiation resistance (radiation loss) and ohmic resistance (damping loss). Using RLC model, the Q-factor can be shown to have little dependence on the resonator geometrical parameters. This seems to suggests that, under the quasi-static assumption on which the LC model is based, the FoM is fairly constant for all the resonator sizes, and has a specific range of values based on the geometrical shape (i.e., U-SRR, Uf-SRR, and *C*_n_). Nevertheless, it should be noted that the RLC model is only accurate when the resonance is far from the interband transition (near-infrared spectrum or longer). In fact, the damping loss decreases in the mid-visible spectrum just before the interband transition^46^, which then leads to a higher Q factor and FoM.

**Figure 7:**
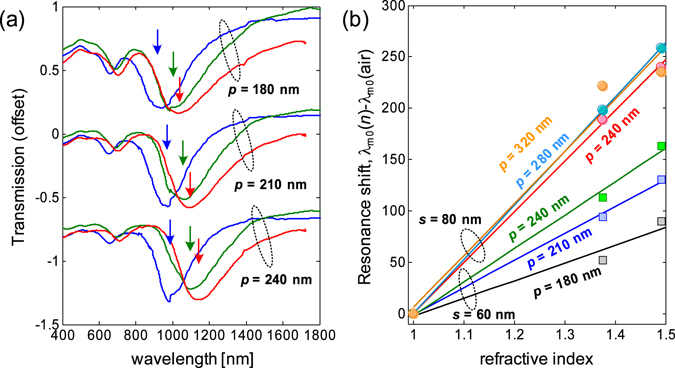
Bulk sensing characteristics of *C*_4_ U-SRR structures. (**a**) Measured transmission spectra of *C*_4_ U-SRRs for *s* = 60 nm, *s* = 80 nm, and *s* = 100 nm under different claddings. The resonance positions for air, isopropyl alcohol solution, and 100-nm thick PMMA are indicated by the blue, green, and red arrows, respectively. (**b**) The *C*_4_ U-SRR bulk sensitivities for different resonator sizes.

The spectral response *C*_4_ U-SRRs under different claddings are presented in Fig. 7, with their experimentally measured (numerically calculated) sensing parameters shown in Table 1 (Table S1). There is a minor difference in the way the Q-factors (Q = λ_R_/Δλ) were deduced in our numerical simulations and experiments. In our simulations, the resonance linewidth (Δλ) is the full-width-at-half maximum (fwhm) of the extinction cross sections, while in the experiments, the resonance linewidth is the fwhm of the spectral contrast. However, it should be noted that, for both calculations and experiments, the Q-factor increases along with the decrease in resonance wavelength (λ_R_) and the spectral contrast [*C*(λ_R_)]. As shown in Table 1, the highest measured Q-factor was *Q* ~ 13 at λ_R_ = 738 nm (1.68 eV) for the 60-nm sized U-SRR. It is important to note that our experimental and calculated Q-factors remain smaller than the intrinsic limit for localized surface plasmons^47^ (Q~20 for 1.6–1.8 eV photon energy), which suggests the validity of our measured and calculated Q-factor values. The highest measured Q-factor is also comparable with the recently reported Q-factor of gold nanorod enhanced by encapsulated annealing^48^, which illustrates the good fabrication quality of our metal nanostructures. The Γ ∝ λ_R_ and FoM ∝ Q relations are evident from our numerical simulations (Table S1), in good agreement with the LC model (see Fig. S2, Supplementary Information). However, only the Γ ∝ λ_R_ relation was observed in our experiments (Table 1). In addition, the FoM decreases at shorter resonance, in contrast to the LC model and FDTD calculations. This is attributed to the resonance broadening from inter-resonator coupling. This is verified in our numerical simulations, where the resonance for the periodic structures appears to be broader than the resonance for isolated structure. The other likely factor is the $\propto 1/\sqrt{{\varepsilon }_{d}}$ dependence in the sensitivity, which suggests that a lower substrate refractive index gives higher sensitivity. This is consistent with the fact that our gold nanostructures were fabricated on indium-tin-oxide (ITO) coated glass, which has higher refractive index than that of SiO_2_ (used in numerical simulations). Despite these differences, the calculated and measured FoMs of U-SRR are in reasonable agreements for the most cases.

**Table 1:** Sensing parameters of *C*_4_ U-SRR.

C_4_ *U*-SRR (experiments)							
*s* [nm]	λ_R_ [nm]	C(λ_R_)	T(λ_R_)	R(λ_R_)	*Q*	Γ [nm/RIU]	FoM
60	738.05	0.07	0.42	0.34	13.20	107.93	1.93
70	899.78	0.17	0.50	0.32	9.43	218.45	2.29
80	1075.08	0.18	0.56	0.28	6.91	348.94	2.24
90	1165.72	0.14	0.55	0.25	7.00	452.65	2.71
100	1349.77	0.13	0.61	0.24	7.50	515.93	2.87

**Figure 8:**
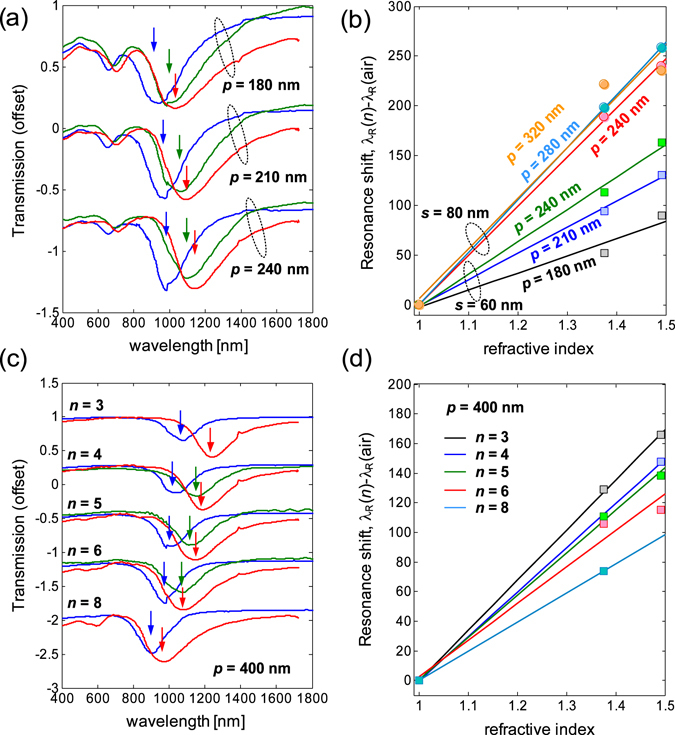
Bulk sensing characteristics of gammadion (Uf-SRR) and star (*C*_n_) structures. (**a**) Measured transmission spectra of Uf-SRR structures (s = 60 nm) coated by different claddings at different periodicities. (**b**) The Uf-SRR bulk sensitivities as a function of periodicity and size. (**c**) Measured transmission spectra of *C*_n_ structures for s = 100 nm and *p* = 400 nm under different claddings. (**d**) The *C*_n_ bulk sensitivities for s = 100 nm at *p* = 400 nm. The resonance positions for air, isopropyl alcohol solution, and 100-nm thick PMMA are indicated by the blue, green, and red arrows, respectively. Some measurements of isopropyl alcohol (for *n* = 8) were not available due to the evaporative nature of the liquid.

The spectral characteristics of Uf-SRR and *C*_n_ structures for different cladding are shown in Fig. 8, with their measured (calculated) sensing parameters of Uf-SRR and *C*_n_ structures presented in Table 2 (Table S2). These structures were fabricated at different periodic spacing with the purpose of studying the inter-resonator coupling. The spectral contrast of the rotationally symmetric structures is much higher than that of *C*_4_ U-SRRs, but their Q-factors are generally lower (Table 2). This is an expected outcome from the conductive coupling of longitudinal plasmons from the constituent nanorods. As the lattice periodicity is increased, we observed a slight increase in Q-factor and resonance wavelength (λ_R_) for Uf-SRR and *C*_n_ structures. This is attributed to a decrease of inter-resonator coupling, which is dominated by transverse dipolar coupling. For the Uf-SRR, the effect of inter-resonator coupling to the sensitivity is illustrated in *s* = 60 nm as the periodicity is varied from 180 nm (*p* = 3 *s*) to 240 nm (*p* = 4 *s*), where the sensitivity and FoM increase from ~172 nm/RIU to ~325 nm/RIU and from ~0.81 to ~1.89, respectively. However, this effect is not observed in *s* = 80 nm, where both Γ and FoM slightly decrease as the period is increased from 280 nm to 320 nm. The sensing parameters for *s* = 100 nm are not available as they were beyond the measurement range of our equipment. Despite having ~3x spectral contrast enhancements, the FoM of the Uf-SRR structure is still lower than C_4_ U-SRR (1.93 < FoM < 2.87). For the *C*_n_ structures, on the other hand, we have 1.47 < FoM < 1.73 (for *p* = 300 nm) and 1.16 < FoM < 1.83 (for *p* = 400 nm), with sensitivities comparable to those of Uf-SRRs and *C*_4_ U-SRRs for the same wavelength ranges. We observed a drop in the sensitivity as the number of arms is increased from *n* = 3 to *n* = 8. This is consistent with the Γ ∝ λ_R_ relation from the LC model, as increasing number of arms translates to shorter resonance wavelength. In terms of the inter-resonator coupling effect, we observed a drop of sensitivity as the periodicity is increased for the *C*_n_ structures. This is in contrast with the Uf-SRR structure, which has higher sensitivity for larger periodicity.

**Table 2:** Sensing parameters of Uf-SRR and *C*_n_ structures.

Gammadion (*Uf*-SRR)								
*s* [nm]	*p* [nm]	λ_R_ [nm]	C(λ_R_)	T(λ_R_)	R(λ_R_)	*Q*	Γ [nm/RIU]	FoM
60	180	944.09	0.40	0.20	0.58	4.45	172.10	0.81
60	210	964.16	0.51	0.23	0.49	5.60	263.21	1.53
60	240	998.57	0.54	0.28	0.41	5.80	325.23	1.89
80	240	1311.06	0.55	0.21	0.50	3.67	493.00	1.38
80	280	1343.32	0.50	0.31	0.40	4.29	527.44	1.68
80	320	1380.18	0.44	0.40	0.32	4.34	507.06	1.60
100	300	1706.91	0.44	0.31	0.38	—	—	—
100	350	1736.41	0.41	0.42	0.31	—	—	—
100	400	1817.51	0.34	0.55	0.31	—	—	—
***C***_**n**_ **structure**, ***s*** = **100** **nm**, ***p*** = **300** **nm**								
***N***	**λ**_**R**_ [**nm**]	**C**(**λ**_**R**_)	**T**(**λ**_**R**_)	**R**(**λ**_**R**_)	***Q***	**Γ** [**nm/RIU**]	**FoM**	
3	1071.43	0.47	0.50	0.30	4.99	335.16	1.56	
4	1025.35	0.54	0.42	0.35	4.78	371.56	1.73	
5	1002.30	0.60	0.34	0.41	4.68	333.19	1.56	
6	960.83	0.64	0.26	0.45	4.56	319.76	1.52	
8	864.06	0.58	0.23	0.53	4.30	295.73	1.47	
***C***_**n**_ **structure**, ***s*** = **100** **nm**, ***p*** = **400** **nm**								
***N***	**λ**_**R**_ [**nm**]	**C**(**λ**_**R**_)	**T**(**λ**_**R**_)	**R**(**λ**_**R**_)	***Q***	**Γ** [**nm/RIU**]	**FoM**	
3	1080.65	0.34	0.65	0.24	5.84	338.57	1.83	
4	1039.17	0.40	0.58	0.27	5.43	299.67	1.57	
5	1016.13	0.46	0.50	0.30	5.35	285.47	1.50	
6	974.65	0.59	0.35	0.34	5.84	246.96	1.48	
8	900.92	0.56	0.32	0.41	5.29	197.15	1.16	

**Figure 9:**
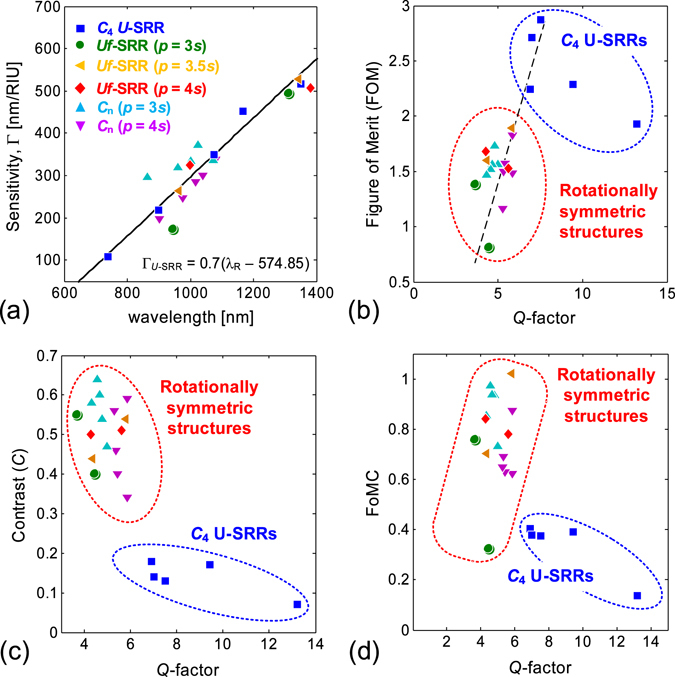
Sensing performances of *C*_4_ U-SRR, Uf-SRR, and *C*_n_ structures based on unpolarized light. (**a**) Sensitivity as a function of resonance wavelength, (**b**) Figure-of-Merit as a function of Q-factor, (**c**) Spectral contrast as a function of Q-factor, and (**d**) Modified Figure-of-Merit (FoMC) as a function of Q-factor. The solid line in (**a**) is the linear fitting of Γ_SRR_ (λ_R_), while the dashed line in (**b**) is a guide line depicting the FoM ∝ Q relation. The shaded areas in (**b**)–(**d**) group the *C*_4_ U-SRRs and rotational symmetric structures (Uf-SRR and *C*_n_).

For completeness, we show in Fig. 9 the relations between different sensing parameters for all the structures considered in this work. As shown in Fig. 9a, the sensitivities of all the structures exhibit a linear dependence on the resonance wavelength, with the solid line depicting the linear fit of the sensitivity of the *C*_4_ U-SRR. The same relation holds for the Uf-SRR and *C*_n_ structures, but with vertical offsets from the solid line. Numerical calculations have been performed to confirm this (Fig. S2a), with those of *C*_n_ and U-SRRs are only vertically offset from each other due to geometrical factors. A similar linear dependence is also found for self-assembled gold nanoparticles^49, 50^. Figure 9b presents the figures of merit of all the structures, showing that the *C*_4_ U-SRRs evidently exhibit the highest FoM among the rotational symmetric structures. The FoM ∝ Q relation seems to apply only for lower Q-factors in our experiments (indicated by the dashed line), but this relation appears to be true for all Q-factors in our numerical simulations (Fig. S2b).

The inverse relation between the spectral contrast and the Q-factor is clearly illustrated in Fig. 9c, where Uf-SRR and *C*_n_ structures display ~3x higher spectral contrast than do *C*_4_ U-SRRs. As both spectral contrast and FoM depend on Q-factor, it is thus natural that higher contrast is achieved at the expense of reduced FoM. An optical cavity system with high FoM likely suffers from low spectral contrast since the effective cavity loss would be amplified by the high-Q resonance. For this reason, it is necessary to include the spectral contrast into the figure of merit, and then define a modified figure of merit (FoMC) as ${\rm{FoMC}}={\rm{FoM}}\times C({\lambda }_{R})$. Using FoM = ΓQ/λ_R_, coupled with Γ ∝ λ_R_ (Fig. 9a) and *C*(λ_R_) ∝ 1/Q (Fig. 9c), it can be shown that FoMC = (Γ/λ_R_)[C(λ_R_)Q] is nearly constant, making FoMC a more general figure-of-merit than the typical FoM, which still have dependence on the Q-factor. The FoMC for all the structures are shown in Fig. 9d. The *C*_4_ U-SRR exhibits FoMCs in the range of 0.13 < FoMC < 0.4, which are lower than those of Uf-SRR (0.32 < FoMC < 1.01) and *C*_n_ (0.62 < FoMC < 0.97).

In summary, we have investigated various rotationally symmetric metal nanostructures for realizing sensing platform with strong optical response and robust mode excitation under unpolarised light. We have presented the resonance properties of Uf-SRR and *C*_n_ structures, which are expressible as a superposition of the longitudinal plasmons from their constituent nanorods. We have shown that the nanorods can be carefully positioned to yield desired optical responses while still preserving the electric characteristics of the constituent nanorods. Introducing internal rotational symmetry (in Uf-SRR) into a system with pure external rotational symmetry (in *C*_4_ U-SRR) has been observed to further enhance the reflectance by ~2x and spectral contrast by ~3x. The role of increasing internal rotational symmetry is further studied in *C*_n_ structures, showing ~2x enhancement in optical response as the rotational symmetry is changed from three-fold (*n* = 3) to eight-fold (*n* = 8). The sensing performance of rotationally symmetric structures has been studied both numerically and experimentally, and the linear dependence of the sensitivity on the resonance wavelength and the linear dependence of FoM on the Q-factor have been analytically derived based on the *LC* model. We have shown that all the structures exhibit comparable sensitivities, and that the stronger spectral contrast is achieved at the expense of decreasing FoM and Q-factor. The highest FoM is found to be with *C*_4_ U-SRR structures, which is also associated with the highest achievable Q-factor among all the nanostructures. The inter-resonator coupling is found to contribute differently in Uf-SRR and *C*_n_ structures, where decreasing (increasing) inter-resonator coupling give rise to higher (lower) sensitivity for Uf-SRR (C_n_) structures. Finally, taking into account the trade-off between spectral contrast and Q-factor, we have proposed a modified figure-of-merit (FoMC) which we believe serves as a more general criterion for evaluating the performances of a sensing platform. Based on this criterion, we demonstrate that the rotationally symmetric structures (Uf-SRR and *C*_n_) exhibit ~2–3x higher FoMC than do the *C*_4_ U-SRRs.

## Methods

### Nanofabrication

The gammadions, star-shaped nanostructures, and the split ring resonators were all fabricated on the same indium tin oxide (ITO) coated glass substrate by electron beam lithography followed by a standard gold lift-off process. The e-beam patterning was carried out at 360–400 pC/cm^2^ exposure dose based on 20 keV beam energy and 20–30 pA beam current (Raith e_LiNE). Each device covers 100 × 100 μm^2^ footprint, surrounded by markers denoting its orientation. The physical metal deposition was done by e-beam evaporation (Edwards 306), where 2-nm thick titanium was first deposited (at 0.01 nm/s) before 30-nm thick gold deposition (at 0.05 nm/s) took place. The samples were then immersed in warm *n*-methylpyrrolidone solution (Remover PG) for 10–20 mins for lift-off pattern transfer.

### Device characterization

The measurements were carried out by CRAIC spectrophotometer using unpolarized broadband source (UV-Vis-NIR) at normal incidence. The light signal from 80 µm × 80 µm aperture was collected through 15x Objective lens (NA = 0.28), which was then normalized with the background spectrum of ITO glass (for transmission) or of gold pad (for reflection). For sensing performance evaluations, different refractive indices were introduced by thin film coating (90 nm thick PMMA, Δ*n* ~ 0.49) and dropping isopropanol (Δ*n* ~ 0.374), while the sensitivity was deduced by a standard linear fitting.

### Numerical calculation

We performed finite difference time domain simulations (FDTD solutions, *Lumerical* Inc) to calculate the optical responses of the rotationally symmetric structures. The scattering properties were calculated using total-field-scattered-field module, where the inward and outward power flows were calculated to deduce absorption (σ_abs_) and scattering (σ_scat_) cross sections. The extinction cross section (σ_ext_) is σ_ext = _σ_abs_ + σ_scat._ The gold permittivity was based on Johnson-Christy model, while the substrate is glass.

## Electronic supplementary material

Supplementary Information
