# Supplementary material for: Polarization invariant plasmonic nanostructures for sensing applications
Source: Sci Rep. 2017 Aug 8;7:7539. doi: 10.1038/s41598-017-08020-y (PMC5548906; doi:10.1038/s41598-017-08020-y)
Supplement: Supplementary file 1 — Supplementary Information [file 41598_2017_8020_MOESM1_ESM.doc]

Supporting Information

Polarization-robust plasmonic nanostructures for sensing applications

Landobasa Y. M. Tobing^1^, Geat-Yee Goh^1^, Aaron D. Mueller^1^, Lin Ke^2^, Yu Luo^1^, Dao-Hua Zhang^1*^

*Corresponding Author: EDHZhang@ntu.edu.sg

1. **Polarization responses of C_n_ structure**

Figure S1. Mapping of the resonance wavelength (λ_R_) for different incident polarization angles. The polarization invariance is clearly observed for even number of arms. The wavelength variation exists for odd *n* due to the opposite conduction current along the gold nanorod as a result of competing magnetic and electric resonance processes. Systematic blue shift is also observed as the *n* is increased from *n* = 3 to *n* = 8 (except for *n* = 4 due to the reason explained in the main text). In this simulation, the arm length (s) and the nanorod width (*w*) is fixed to *s* = 70 nm and *w* = 25 nm, respectively.

1. **Calculated sensing performances of U-SRR, Uf-SRR, and Cn**

Figure S2. Calculated sensing performances for U-SRR, Uf-SRR, and Cn structures: (a) Sensitivity vs. λ_R_, and (b) Figure-of-Merit vs. Q-factor. The Γ  λ_R_ and FoM  Q relations are clearly observed. The effect of periodicity is investigated by setting the period to be *p* = 4s (for Uf-SRR and U-SRR) and *p* = 400 nm (for *C*_n_ structures). Here, *p* = 4*s* is chosen for U-SRR because the SRR does not interact with its immediate neighbours in *C*_4_ configurations because they are orthogonal to each other. The feature width of all structures are set to *w* = 25 nm. The linear relations in all figures are indicated by the dashed lines. For Γ(λ_R_), it is interesting to note that U-SRR, Uf-SRR, and Cn exhibit the same slope, with the Cn vertically offset from U-SRR and Uf-SRR. To make this more visible, we present isolated Cn structure with s = 70 nm.

Table S1. Calculated sensing performances of isolated and periodic U-SRRs.

|  | Isolated *U*-SRR (calculated) | | | | Periodic *U*-SRR (p = 4s) (calculated) | | | |
| --- | --- | --- | --- | --- | --- | --- | --- | --- |
| s (nm) | λ_R_ (nm) | Γ (nm/RIU) | Q | FoM | λ_R_ (nm) | Γ (nm/RIU) | Q | FoM |
| 40 | 828.47 | 523.56 | 19.42 | 12.28 | 824.54 | 520.08 | 11.18 | 7.05 |
| 50 | 1100.72 | 605.66 | 5.80 | 3.19 | 1099.88 | 618.30 | 4.65 | 2.62 |
| 60 | 1194.93 | 645.16 | 5.44 | 2.94 | 1198.39 | 664.78 | 4.45 | 2.47 |
| 70 | 1192.03 | 668.86 | 5.46 | 3.06 | 1181.22 | 648.85 | 4.59 | 2.52 |
| 80 | 1323.96 | 697.83 | 5.03 | 2.65 | 1307.60 | 616.19 | 4.37 | 2.06 |
| 90 | 1433.05 | 764.32 | 4.77 | 2.54 | 1416.82 | 742.59 | 4.21 | 2.21 |
| 100 | 1568.20 | 815.01 | 4.53 | 2.35 | 1538.71 | 812.11 | 3.98 | 2.10 |

Table S2. Calculated sensing performances of isolated and periodic Uf-SRRs *C*_n_ strucrtures.

|  |  |  |  |  |  |  |  |  |
| --- | --- | --- | --- | --- | --- | --- | --- | --- |
|  | Isolated Uf-SRR (calculated) | | | | Periodic Uf-SRR (p=4s) (calculated) | | | |
| s [nm] | λ_R_ [nm] | Γ [nm/RIU] | Q | FOM | λ_R_ [nm] | Γ [nm/RIU] | Q | FOM |
| 40 | 1053.46 | 586.57 | 5.60 | 3.12 | 999.19 | 628.04 | 3.60 | 2.26 |
| 50 | 1191.59 | 650.95 | 5.04 | 2.75 | 1133.53 | 696.77 | 3.64 | 2.24 |
| 60 | 1351.38 | 712.05 | 4.73 | 2.49 | 1374.65 | 761.95 | 3.60 | 2.00 |
| 80 | 1733.87 | 958.66 | 7.57 | 4.19 | 1682.26 | 872.15 | 3.70 | 1.92 |
| 100 | 1903.23 | 1049.37 | 8.19 | 4.52 | 1974.88 | 921.66 | 3.78 | 1.76 |

|  |  |  |  |  |  |  |  |  |
| --- | --- | --- | --- | --- | --- | --- | --- | --- |
| Star-shaped nanostructures, s = 100 nm (calculated) | | | | | | | | |
|  | Isolated | | | | Periodic (p = 400 nm) | | | |
| N | λ_R_ [nm] | Γ [nm/RIU] | Q | FOM | λ_R_ [nm] | Γ [nm/RIU] | Q | FOM |
| 3 | 1370.59 | 602.50 | 7.23 | 3.18 | 1351.04 | 584.60 | 5.78 | 2.50 |
| 4 | 1322.53 | 607.77 | 7.13 | 3.28 | 1281.68 | 576.17 | 5.50 | 2.47 |
| 5 | 1327.38 | 601.45 | 6.85 | 3.10 | 1293.43 | 554.05 | 5.08 | 2.18 |
| 6 | 1325.80 | 551.94 | 6.21 | 2.59 | 1276.15 | 559.32 | 4.70 | 2.06 |
| 8 | 1303.48 | 568.80 | 5.76 | 2.51 | 1220.56 | 576.17 | 3.96 | 1.87 |
